# Stereotactic prostate radiotherapy with or without androgen deprivation therapy, study protocol for a phase III, multi-institutional randomized-controlled trial

**DOI:** 10.1259/bjro.20220032

**Published:** 2022-11-29

**Authors:** Marco Lorenzo Bonù, Alessandro Magli, Davide Tomasini, Francesco Frassine, Domenico Albano, Stefano Arcangeli, Alessio Bruni, Stefano Ciccarelli, Martina De Angeli, Giulio Francolini, Ciro Franzese, Paolo Ghirardelli, Luigi Grazioli, Andrea Guerini, Andrea Lancia, Giulia Marvaso, Matteo Sepulcri, Luca Eolo Trodella, Vittorio Morelli, Andrea Georgopulos, Anastasiya Oleksandrivna Domina, Lorenzo Granello, Eneida Mataj, Fernando Barbera, Luca Triggiani

**Affiliations:** 1 Department of Radiation Oncology, Istituto del Radio O.Alberti, University of Brescia and Spedali Civili Hospital, Brescia, Italy; 2 Department of Radiation Oncology, AULSS 1 Belluno, Belluno, Italy; 3 Department of Nuclear Medicine, University of Brescia and Spedali Civili Hospital, Brescia, Italy; 4 Department of Radiation Oncology, ASST Monza Ospedale San Gerardo, Monza, Italy; 5 Department of Radiation Oncology, Azienda Ospedaliera Universitaria Policlinico di Modena, Modena, Italy; 6 Department of Radiation Oncology, ASST – Cremona, Cremona, Italy; 7 Department of Radiation Oncology, Fondazione Policlinico Tor Vergata, Rome, Italy; 8 Department of Radiation Oncology, Azienda Ospedaliera Universitaria Careggi, Firenze, Italy; 9 Department of Radiation Oncology, Humanitas Research Hospital, Rozzano, Milano, Italy; 10 Department of Radiation Oncology, Humanitas Gavazzeni Hospital, Bergamo, Italy; 11 Department of Radiology, ASST Spedali Civili di Brescia, Brescia, Italy; 12 Department of Radiation Oncology, Fondazione IRCCS Policlinico, San Matteo, Italy; 13 Department of Radiation Oncology, Istituto Europeo di Oncologia (IEO), Milano, Italy; 14 Department of Radiation Oncology, Istituto Oncologico Veneto IOV-IRCCS, Padua, Italy; 15 Department of Radiation Oncology, Policlinico Universitario Campus Bio-Medico, Roma, Italy

## Abstract

**Objective:**

The therapeutic landscape for localized prostate cancer (PC) is evolving. Stereotactic radiotherapy (SRT) has been reported to be at least not inferior to standard radiotherapy, but the effect of androgen deprivation therapy (ADT) in this setting is still unknown and its use is left to clinical judgment. There is therefore the need to clarify the role of ADT in association with SRT, which is the aim of the present study.

**Methods:**

We present a study protocol for a randomized, multi-institutional, Phase III clinical trial, designed to study SRT in unfavorable intermediate and a subclass of high-risk localized PC. Patients (pts) will be randomized 1:1 to SRT + ADT or SRT alone. SRT will consists in 36.25 Gy in 5 fractions, ADT will be a single administration of Triptorelin 22.5 mg concurrent to SRT. Primary end point will be biochemical disease-free survival. Secondary end points will be disease-free survival, freedom from local recurrence, freedom from regional recurrence, freedom from distant metastasis and overall survival (OS); quality of life QoL and patient reported outcomes will be an exploratory end point and will be scored with EPIC-26, EORTC PR 25, IPSS, IIEF questionnaires in SRT + ADT and SRT alone arms. Moreover, clinician reported acute and late toxicity, assessed with CTCAE v. 5.0 scales will be safety end points.

**Results:**

Sample size is estimated of 310 pts. For acute toxicity and quality of life results are awaited after 6 months since last patient in, whereas, for efficacy end points and late toxicity mature results will be available 3–5 years after last patient in.

**Conclusion:**

Evidence is insufficient to guide decision making concerning ADT administration in the new scenario of prostate ultra-hypofractionation. Hence, the need to investigate the ADT role in SRT specific setting.

**Advances in knowledge:**

The stereotactic prostate radiotherapy with or without ADT trial (SPA Trial) has been designed to establish a new standard of care for SRT in localized unfavorable intermediate and a subclass of localized high risk PC.

## Introduction

### Background

Radiotherapy (RT) for the treatment of localized prostate cancer (PC) is changing. The ProtecT trial showed no differences in terms of overall survival (OS) and progression-free survival (PFS) for patients treated with RT *vs* radical prostatectomy *vs* active monitoring, with a different toxicity profile among the three options.^1^ The observation of a lower than expected α/β ratio in PC cells has led to explore moderate hypofractionation schedules, that are actually considered not inferior to the normofractionated radiation regimes, thanks to the results of the CHHiP, the PROFIT, and the RTOG 0415 trials. Brachytherapy boost plus normofractionated external beam radiotherapy (EBRT) is also an option in this setting of patients.^2–4^

The dramatic technical innovation in recent years allowed to explore feasibility and safety of stereotactic body ablative radiotherapy (SRT) schedules in PC. SRT has the advantage to deliver high-precision, high-dose RT in a short period of time (generally 1 to 8–10 fraction), with a convenience for patients and RT facilities. A broad spectrum of Phase I, II and III clinical trials and a recent meta-analysis showed promising results in terms of local control, biochemical relapse-free survival and relapse-free survival with an acceptable risk for toxicity using a 7-fractions schedule, mainly genitourinary (GU) side-effects. Remarkably the 5-fraction SRT schedule was associated with a slightly worse acute GU toxicity profile and an equal late GU toxicity risk profile in comparison to standard EBRT.^5–7^

The PACE-B non-inferiority, randomized, Phase III trial, comparing the efficacy of 5-fraction SRT schedule with moderately hypofractionated RT for the treatment of low and intermediate risk PC patients in terms of freedom from biochemical recurrence and disease-free survival (DFS) is also ongoing.

Results of HYPO-RT-PC Phase III randomized trial have been recently published, with intermediate and high risk PC patients in SRT arm presenting non-inferior outcomes compared to normofractionation. Androgen deprivation therapy (ADT) was not permitted in both trials, either in the standard arm nor in the experimental one.^6^

### Unfavorable intermediate and high-risk prostate cancer: SRT–ADT dilemma

D’Amico/NCCN risk stratification of disease is an essential strong prognostic factor in PC. Intermediate risk class has been further divided in two subgroups with different prognosis, favorable intermediate and unfavorable intermediate risk. In case of normofractionated and moderate hypofractionated schedules there is evidence that short-term ADT combined with RT improves OS, PFS and biochemical disease-free survival (bDFS). Despite these results, the attitude of clinicians has been slightly different for prostate SRT, with the frequent omission of ADT in the absence of clear and consolidated data in literature; with no clinical trials ongoing to clarify this issue. Such clinical attitude may be motivated by the possible better local control that ablative SRT can guarantee. Nevertheless, emerging evidence from large cohorts studies has shown that unfavorable intermediate risk PC patients treated with SRT present a bDFS not so different from high risk patients. Katz et al published a series of 515 patients undergoing SRT for localized PC. The 47 unfavorable intermediate risk patients presented outcomes quite similar to the high-risk cohort, with a 7 year bDFS of 68 and 65%, respectively. Pattern of relapse was equally distributed among local, distant and biochemical ones, the latter probably reflecting micro-metastatic disease not detectable with current imaging techniques such as Fluoro-Choline positron emission tomography (PET) CT, bone scan and contrast enhanced CT.^8^ The authors conclude that ADT may be of limited benefit in this scenario but given the relatively high rate of relapses in unfavorable intermediate cohort, novel strategies to improve DFS are warranted.

Evidence of the benefit of adding ADT to normofractionated and moderately hypofractionated EBRT for high risk PC patients is even stronger. Despite this, translating the evidence deriving from these studies to ultra-hypofractionation could be tricky, given the different response to high dose per fraction of PC cells.^8,9^ Conversely, the attitude of omitting concurrent ADT in the context of prostate SRT has been clear in recent literature.

Remarkably, in 2016, the randomized Phase III study from Bolla and colleagues demonstrated that combining short-course ADT with normofractionated EBRT improves 5 year bDFS and DFS over RT alone for intermediate and high risk PC patients (hazard ratio 0.52 and 0.63, respectively). Interestingly, there was a statistically significant reduction in 5 year local relapses rate from 6 to 2%, and in the distant metastasis rate from 7.6 to 4.4% (hazard ratio 0.37 in both cases) in the combination arm.^10^

Another reason which may explain the frequent omission of short-term ADT in unfavorable intermediate risk patients eligible for SRT could be related to concerns about the ADT side-effects. Also, in the context of SRT prospective studies identified an increased risk of acute and late GU toxicity in SRT + ADT arm, with a mechanism not completely clear.^11^ Interestingly, Bolla and colleagues in the former randomized, Phase III trial also performed a QoL analysis with HRQOL questionnaires (EORTC QoL questionnaires C30 supplemented with a QLQ-PR25) and showed no clinically relevant differences in HRQOL scores between the groups. As experienced ADT-related symptoms, as well, were more clinically evident and significantly worse in the ADT arm, at month 6 and at year 1, as sexual activity and functioning scales. However, no marked difference was seen between the arms from year 2 onward. Nevertheless, this trial did not consider the impact of ADT on endocrine homeostasis, as reported in a wide body of literature.^12–14^

### Rationale for study design and hypothesis

The present study aims to clarify the role of short-term ADT in the context of unfavorable intermediate and a subclass of high risk PC patients eligible for prostate SRT. Prostate SRT has been endorsed as an option for primary radical treatment of PC. The benefit of ADT is still unknown and previous evidence are insufficient in this setting of patients and the final clinical decision is left to clinical judgement.

### Objective of the trial

For the reasons discussed, it seems to be relevant to propose a randomized, open label, Phase III clinical trial to compare prostate SRT plus 6 months ADT *vs* prostate SRT alone for the treatment of unfavorable intermediate and a subgroup of high risk PC patients, with the aim of demonstrating if SRT + ADT is superior to SRT alone.

### Endpoints of the study

#### Primary end point

Primary end point will be to determine if there is any improvement in bDFS, defined as prostate-specific antigen (PSA) rising ≥2 ng ml^−1^ over PSA Nadir, following the Phoenix criteria for biochemical recurrence of PC (after primary RT) in patients treated with prostate SRT plus ADT (ARM A) compared to patients treated with SRT alone (ARM B).

Study end points are summarized in Table 1.

**Table 1.** Study end points

PRIMARY END POINTS	SECONDARY END POINTS
Determine if there is any improvement in bDFS in patients treated with prostate SRT plus ADT (ARM A) compared to patients treated with SRT alone (ARM B)	DFS FFLR FFRR FFDM OS

bDFS, biochemical disease-free survival; FFDM, freedom from distant metastasis; FFLR, freedom from local recurrence; FFRR, freedom from regional recurrence; OS, overall survival.

All biochemical failures need to be confirmed with a second PSA detection meeting the criteria for failure. In addition, it is now recognized that a benign PSA bounce may be seen in up to 20% of patients after SRT, usually within the first 2 years.^15^

In some cases, the magnitude of the PSA bounce may be considered as high enough for the patient to be incorrectly classified as a PSA failure. To prevent this, for patients receiving SRT, PSA failure before 24 months after the treatment will require three consecutive PSA rises to resulting a clinical diagnosis of failure, or the start of further treatment (androgen deprivation therapy). Over 24 months, the definition of PSA failure for SRT will refer to the Phoenix definition described above (*i.e.* PSA nadir+2 ng ml^−1^).

QoL and patient reported outcomes (PROs) will be an exploratory end point and will be scored with questionnaires EPIC-26, EORTC PR 25, IPSS, IIEF in SRT + ADT and SRT alone arms.

Moreover, Clinician reported Acute Toxicity, assessed with CTCAE v. 5.0 scales and Clinician reported Late Toxicity, assessed with CTCAE v. 5.0 scales will be safety end points.

## Methods and materials

### Trial design and allocation ratio

This is a multicenter, open label, randomized-controlled, Phase III study. Randomization will be 1:1 and will be accounted for minimization with a balancing algorithm for two variables:

- patients age <70 vs ≥70
- unfavorable intermediate risk and a subgroup of PC high risk group

**Figure 1.**
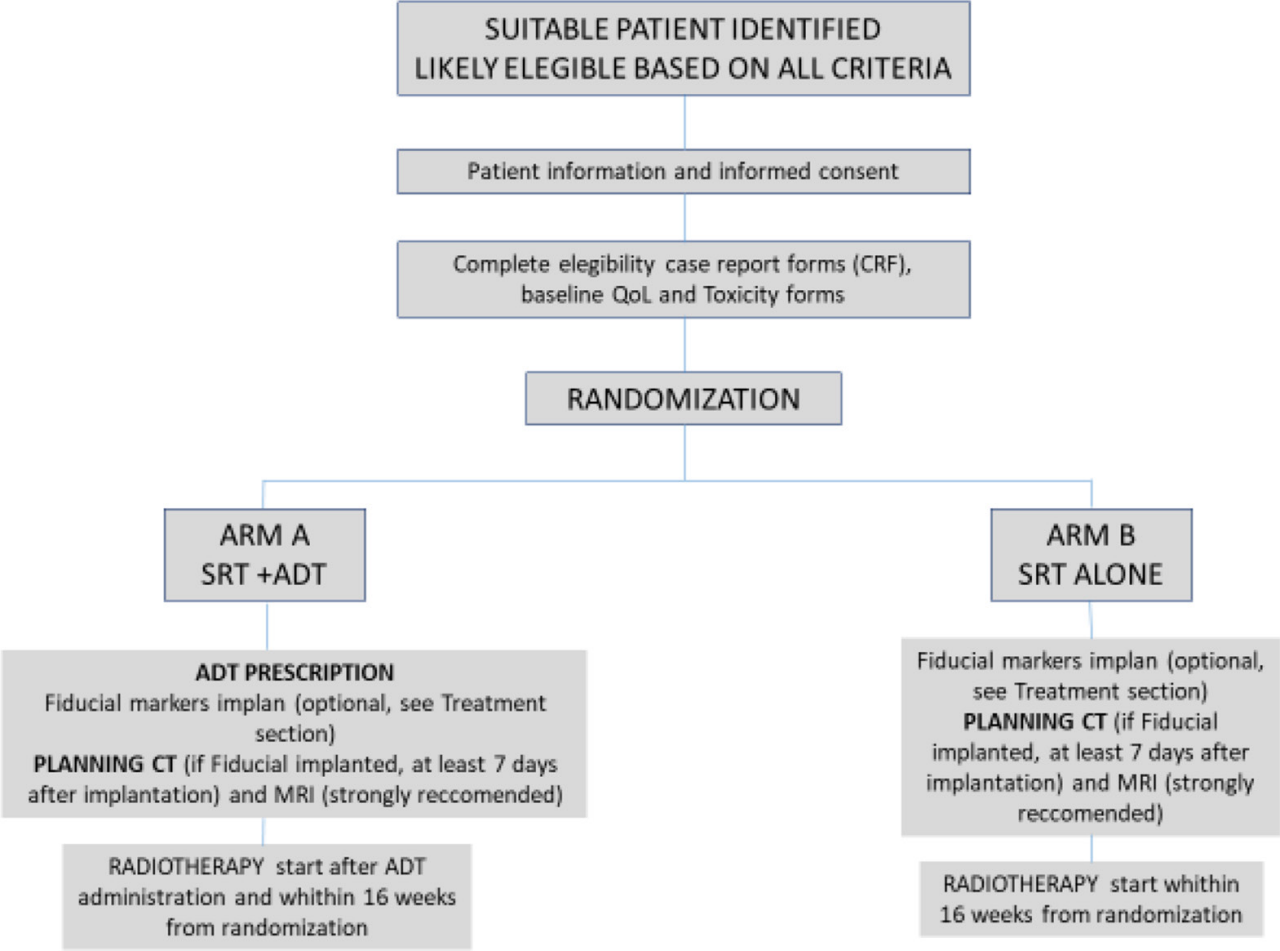
Patient pathway in SPA trial protocol.

In arm A, patients will be treated with prostate SRT to a total dose of 36.25 Gy in 5 fractions (consecutive or alternate days) plus 6 months of ADT consisting in LHRH analog (Triptorelin 22.5 mg). In arm B, patients will be treated with prostate SRT alone to a total dose of 36.25 Gy in 5 fractions (consecutive or alternate days) (Figure 1).

### Key inclusion criteria

- Age 18–80 years old.
- Histological confirmation of prostate acinar adenocarcinoma with a minimum of 10 biopsy cores taken by prostate biopsy
- Prostate protocol MRI for local staging
- Patients belonging to unfavorable intermediate group according to the D’Amico/NCCN risk group classification
- Patients belonging to a subclass of high-risk group according to the D’Amico/NCCN risk group classification:

- ISUP Group 4 (GS 4 + 4, 3 + 5, 5 + 3), or
- cT3a stage, or
- PSA>20

- Eastern Cooperative Oncology Group (ECOG) PS 0–2
- IPSS 0-15
- Prostate volume less than 100 cc
- No pathologic lymph nodes and distant metastasis on PET scan or CT scan plus bone scan

Details about inclusion and exclusion criteria are fully reported in Table 2 and study protocol.

**Table 2.** Study inclusion criteria

KEY INCLUSION CRITERIA	
Age 18–80 years old ECOG PS 0–2 Histological confirmation of prostate acinar adenocarcinoma with a minimum of 10 biopsy cores taken by prostate biopsy Patients belonging to unfavorable intermediate group according to the D’Amico/NCCN risk group classification: Grade Group 3 or/and 2–3 risk factors for intermediate category (PSA 10–20/Grade Group 2–3/cT2a cT2b) or/and Biopsy cores positive ≥50% Patients belonging to a subclass of high-risk group according to the D’Amico/NCCN risk group classification: ISUP Group 4 (GS 4 + 4, 3 + 5, 5 + 3) or cT3a stage or PSA >20	Prostate protocol MRI for local staging Prostate volume less than 100 cc No pathologic lymph nodes and distant metastasis on PET scan or CT scan plus bone scan PSA detection maximum 60 days before randomization IPSS 0–15 Ability of the patient to understand and sign a written informed consent document Ability and willingness to comply with patients reported outcome questionnaires schedule during the study time Contraceptive measures for patients with partners with reproductive potential must be explained

ECOG, Eastern Cooperative Oncology Group; PET, positron emission tomography; PSA, prostate-specific antigen.

### Interventions

### Arm A

Patients in ARM A will be treated with SRT on the prostate (consecutive days or at alternate days to a total dose of 36.25 Gy administered in 5 fractions (7.25 Gy/fraction) plus LHRH analog (Triptorelin 22.5 mg), one administration before SRT. An anti-androgen drug (es. Bicalutamide 50 mg) must be administered daily starting from 7 days before LHRH analog administration to 10 days after to prevent the flare effect.

### Arm B

Patients in ARM B will be treated with SRT on prostate alone at a total dose of 36.25 Gy administered daily or every other day in 5 fractions (7.25 Gy/fraction). SRT treatment planning procedure and ADT prescription are diffusely explained in study protocol.

### Source of the study drug

Triptorelin 22.5 mg and Bicalutamide 50 mg will be provided by the sponsor institution and delivered by express courier to the pharmacy of each Institution.

Bicalutamide will be directly delivered by the medical staff after adequate and comprehensive instructions (50 mg daily, starting from 7 days before LH–RH analog to 10 days after). Triptorelin will be administered directly by nurses or physicians as a single intramuscular injection at least the day before SRT starting, with an encorauged time of administration 90 days before SBRT for cytoreductive purposes (not mandatory).

### Outcome measures and study timeline

Concerning the timing of the study, the accrual time will be 3 years and the follow-up (FU) length will be 5 years.

Patients will be screened before treatment beginning concerning performance status (PS), Testosterone, digital rectal examination (DRE), PRO (IPSS, IIEF), QoL (EPIC 26 and EORTC PR 25). Screening will exploit an online dedicated platform (www.trialspa.it). After meeting every eligibility criteria, a centralized randomization procedure will be automatically performed with a dedicated in-house software.

RT will be started within 16 weeks from randomization.

ADT will be administered form 12 weeks before RT to immediately before RT.

Evaluation will be daily performed during SRT and the last day of treatment to assess acute toxicity (CTCAE 5.0). During the last day of treatment also PRO and QoL will be scored.

Subsequently, patients will be assessed at 1 months after SRT for PS, DRE, acute toxicity, PRO and QoL.

Afterwards, 3 months after SRT completion, PSA, PS and DRE will be assessed together with toxicity PRO and QoL. For the first year of follow-up, patients will be evaluated every 3 months.

**Figure 2.**
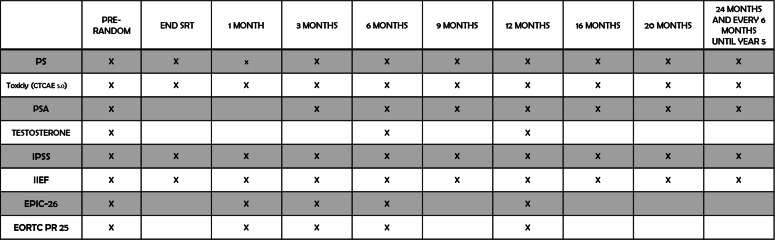
Pre-randomization and follow-up assessment of PSA, testosterone, toxicity, PRO e QoL questionnaires in details. PRO, patient reported outcome; PSA, prostate-specific antigen; QoL, quality of life.

After 12 months from the end of treatment, the FU will be every 4 months. Instead, it will be scheduled every 6 months starting 24 months after the end of treatment (Figure 2).

### Patient and public involvement

Patients or the public were not involved in the design, or conduct, or reporting, or dissemination plans of our research.

## Statistical considerations

### Sample size determination

In the context of normofractionated, dose-escalated PC treatment, 5 years bDFS obtained with ADT and without ADT are reported to be 84 and 69%. Due to the lack of standardized use of ADT in SRT patients, we can estimate from preliminary data on intermediate- and high risk patients a difference in bDFS of 10% at 5 years. In ARM A (SRT +ADT), we expect a bDFS of 95% at 5 years, whereas in ARM B (SRT alone), we expect a bDFS of 85% at 5 years. Considering an α error of 0,05 (2-tailed) and a 1-β of 80%, a study sample of 310 patients, 155 patients for each arm will be necessary to detect a difference of at least 10% in the two groups with a power of 83.9%.

Freedom from biochemical/clinical progression will be compared by proportions, respectively of 85 and 95% of bDFS expected in two arms, according to the sample size hypothesized above. Estimates of event rates will be calculated using the Kaplan–Meier method. The Cox proportional hazard model will be used to adjust for risk group and important known prognostic factors. Gathered data will be analyzed using SPSS^®^ v. 26.0 software (IBM^®^).

### Acute and late toxicity

Acute and late toxicity will be summarized by the proportions experiencing grade ≥2 side-effects with comparisons made (where appropriate) using χ^2^ based tests or Fisher’s exact test if expected cell frequencies will be less than 5.

### Randomization

The study will employ a 1:1 randomization between Arm 1: Arm 2, and will be accounted for minimization with a balancing algorithm for two variables:

- age <70 ≥70,
- unfavorable intermediate- and high risk group.

Patients will be randomized in permuted blocks, with the size of the blocks known only to the statistician. The randomization sequence is known only to the statistician and uploaded into a restricted-access database (www.trialspa.it) housed on secure hospital servers at University of Brescia and Spedali Civili Hospital. The Local PI, before enrolling a patient, will access with a personal login ID and password the internet site to perform the screening procedure. If successfully completed, an automatic allocation for the patient ARM will be provided.

### Analysis plan

Patients will be analyzed in the groups to which they are assigned (intention-to-treat). De-identified data (except for study number and initials) will be registered on the dedicated website requiring personal ID and password.

Study co-ordinators at the sponsor institute will perform data checks throughout the trial period and will inform participating centers if necessary.

Moreover, a monitoring committee will be provided with three visits per/year the first and second year of the study and two times per/year the third year of the study to ensure quality of data.

The primary analysis concerning QoL and PRO in both arms will be at 12 weeks, whereas 6 months and 12 months will be time points of specific interest. Standard algorithms will be used to derive scores from and handle missing data in questionnaires (IPSS, IIEF-5, EPIC-26 and EORTC-PR25). Treatment groups will be compared at individual time points and analyses to account for the longitudinal nature of the data (generalized estimating equations) may be used.

The primary analysis of freedom from biochemical/clinical progression will be event driven unless the Control Committee and Trial Steering Committees agree that the analysis performed before the target number of events is reached would be mature and robust to have the potential to influence clinical practice.

### Interim analysis

Due to the lack of data in the context of ADT combined with prostate SRT for the treatment of PC, an interim analysis after a 50% accrual of sample size will be planned. The aim of this procedure will be to verify the subsistence of the previously reported statistical hypothesis. A sample size re-estimation will be performed in case interim results will differ from our estimates more than 15%.

## Discussion

In the context of localized PC, the evidence of a safe, effective and more convenient treatment will move the clinical and patient’s choice toward SRT. Advances in image guidance such as MRI-guided RT is a promising approach to increase the therapeutic index in this context.^16^ Remarkably, several recent studies, including clinical trials, have tested SRT efficacy on localized PC omitting ADT.^5–7,15,17^

Of note, ADT is widely recognized to improve outcomes in combination with standard and moderate fractionated RT for a subclass of intermediate- and high risk PC patients. Biological rationale of combining RT and ADT were reported in literature,^10^ although the role of ADT in the context of high dose-per-fraction schedule is still uncertain.^18^ Therefore, ADT effect in combination with normofractionated and hypofractionated RT is not clearly movable to ultra-hypofractionation.^8,9^

Recently, in high risk patients, the SHARP consortium prospectively evaluated 344 treated patients, with the aim to explore the efficacy and toxicity of SRT in the context of high risk PC. 72% of patients received ADT (investigator’s choice), with a median duration of 9 months. At univariate analysis, 4 years bRFS resulted significantly greater among patients treated with concurrent ADT but 4 years DMFS was not significantly different.^11^ Given the non-randomized nature of the studies included, selection bias may have influenced the choice to administer ADT. As a consequence, such variable did not enter in multivariable model. Differently, Jackson et al in a meta-analysis of over 6000 pts treated on prospective studies did not found any advantage for ADT use on bDFS, Remarkably, in both studies, ADT use was administered at investigator’s choice and therefore caution must be exercised in the interpretation of these results.

Regarding toxicity, multivariable logistic regression for acute and late toxicity showed that ADT use and 8 Gy per-fraction schedule were associated with higher risk of GU Grade ≥ 3. The physiopathogenesis of increased toxicity with ADT use is unclear. One of the hypothesis concerning both GU and gastrointestinal (GI) side-effects consists in the possible downstaging and shrinking effect determined by ADT that could contribute to an higher than expected dose to critical organs such as bladder and rectum.^11,19^

The optimal dose for prostate SRT is still unknown. Levin-Epstein et al retrospectively compared crude biochemical control, bRFS and ablation rate of four different ultra-hypofractionated schedules in 1908 men. Authors show a hypothesis of superiority of 40 Gy in 5 fractions in terms of crude biochemical recurrence. Interestingly, this study showed no difference in terms of 5 years bRFS, and all the schedules obtained brilliant results in term of bRFS (all showing at least 93% bRFS probability at 5 years, without ADT). Moreover, no data on local control and toxicity were reported.^20^

Concern about the limited therapeutic window of prostate SRT was suggested also by a systematic review and meta-analysis of 6000 pts, where an impact on bRFS of more aggressive schedule was proposed, but a significantly increase in GU Grade ≥ 3 toxicity was also registered. 36,25 Gy in 5 fractions showed similar GU and GI toxicity rates compared to moderately hypofractionated RT.^5^ In terms of efficacy, available studies reported excellent bRFS rates, also without ADT, in unfavorable intermediate and high risk pts.^11,20^

Given these hypotheses and the lack of high quality randomized data directly testing the eventual superiority of a specific SRT regimen, 36.25 Gy in 5 fractions has been the schedule of choice for our trial.

In this scenario, clarification of the contribution of ADT in addition to prostate SRT is clearly needed. We are confident that the present multi-institutional, randomized-controlled trial will provide clinicians with reliable evidence to support treatment decisions. The primary analysis of QoL in both arms will be at 12 weeks, whereas 6 months and 12 months will be time points of specific interest, allowing to obtain in a relatively short time valuable information regarding the tolerability of the SRT + ADT association. The main limitation of the study is that the statistical hypotheses of the expected difference in bDFS between the two groups could be rejected, due to the lack of data in this context (as this is still an unexplored scenario). For this reason, an interim analysis after an accrual of 50% of the expected patients has been planned, and then a sample size re-estimation will be performed whether interim results differ from our estimates by more than 15%. Another limitation of this trial, is the possibility to use both prostate-specific membrane antigen (PSMA)-PET/CT and conventional imaging (99mTc bone scan and CT scan) for the initial staging of disease. Recent publications reported a higher accuracy of PSMA-PET/CT than conventional imaging in the initial staging assessment of males with newly diagnosed, high risk PC. In fact, PSMA-PET/CT has proven to have higher specificity and positive-predictive value in detecting sites of disease that are not visible in conventional imaging.^21,22^ Such findings may suggest the potential creation of two further subgroups in our study: patients with non-metastatic, high risk PC submitted to conventional staging of disease, and patients with non-metastatic, PSMA-negative, high risk PC. A post-hoc subgroup analysis of these two cohorts of patients could demonstrate the possible clinical implication of these two different staging modalities, and further clarify the impact of next-generation imaging in the management of high risk PC.

## Conclusions

SRT is considered at least not inferior to standard RT in the setting of unfavorable intermediate- and high risk PC. Translation of evidence of efficacy from moderate hypofractionation could not be correct given the unique tumor response to ultra-hypofractionation. The effect of ADT in such new scenario is still unknown and its use is left to clinical judgment. The SPA trial has been designed to establish a new standard of care for SRT in localized unfavorable intermediate and a subclass of localized high risk PC.
